# Supplementary material for: Experimental demonstration of generalized quantum fluctuation theorems in the presence of coherence
Source: Sci Adv. 2025 May 30;11(22):eadq6014. doi: 10.1126/sciadv.adq6014 (PMC12124356; doi:10.1126/sciadv.adq6014)
Supplement: Supplementary file 1 — Sections S1 to S3 Figs. S1 to S12 Table S1 References [file sciadv.adq6014_sm.pdf]

Science Advances
AAAS

Supplementary Materials for

Experimental demonstration of generalized quantum fluctuation theorems in the presence of coherence

Hui Li *et al.*

Corresponding author: Lijian Zhang, lijian.zhang@nju.edu.cn; M. S. Kim, m.kim@imperial.ac.uk

Sci. Adv. **11**, eadq6014 (2025)
DOI: 10.1126/sciadv.adq6014

This PDF file includes:

Sections S1 to S3
Figs. S1 to S12
Table S1
References

S1 Quantum channels for testing quantum fluctuation theorems

S1.1 Generic quantum channel involving dissipation

We consider a general quantum channel that involves both rotation and dissipation of the following form:

$$\mathcal{N}(\hat{\rho}) = p\hat{\rho} + (1-p)[(1-s)\mathcal{R}_y^{\pi/2}(\hat{\rho}) + s\mathcal{D}_{\kappa,\lambda}(\hat{\rho})]. \tag{S1}$$

The dissipation channel is described as

$$\begin{aligned}\mathcal{D}_{\kappa,\lambda}(\hat{\rho}) = \kappa \begin{pmatrix}1 & 0\\ 0 & 0\end{pmatrix} \hat{\rho} \begin{pmatrix}1 & 0\\ 0 & 0\end{pmatrix} + (1-\kappa) \begin{pmatrix}0 & 0\\ 1 & 0\end{pmatrix} \hat{\rho} \begin{pmatrix}0 & 1\\ 0 & 0\end{pmatrix}\\ + \lambda \begin{pmatrix}0 & 1\\ 0 & 0\end{pmatrix} \hat{\rho} \begin{pmatrix}0 & 0\\ 1 & 0\end{pmatrix} + (1-\lambda) \begin{pmatrix}0 & 0\\ 0 & 1\end{pmatrix} \hat{\rho} \begin{pmatrix}0 & 0\\ 0 & 1\end{pmatrix},\end{aligned} \tag{S2}$$

where the corresponding Kraus operators are

$$\hat{K}_{00}^{\mathcal{D}} = \begin{pmatrix}\sqrt{\kappa} & 0\\ 0 & 0\end{pmatrix}, \quad \hat{K}_{01}^{\mathcal{D}} = \begin{pmatrix}0 & 0\\ \sqrt{1-\kappa} & 0\end{pmatrix}, \quad \hat{K}_{10}^{\mathcal{D}} = \begin{pmatrix}0 & \sqrt{\lambda}\\ 0 & 0\end{pmatrix}, \quad \hat{K}_{11}^{\mathcal{D}} = \begin{pmatrix}0 & 0\\ 0 & \sqrt{1-\lambda}\end{pmatrix}. \tag{S3}$$

Meanwhile, we construct the rotation part of the channel by mixing two y-rotations,

$$\mathcal{R}_y^{\pi/2}(\hat{\rho}) = \frac{1}{2}[R_y(\pi/2)\hat{\rho}R_y^\dagger(\pi/2) + R_y^\dagger(-\pi/2)\hat{\rho}R_y^\dagger(-\pi/2)], \tag{S4}$$

where the corresponding Kraus operators are

$$\hat{K}_{\pi/2}^{\text{Rot}} = \frac{1}{\sqrt{2}}R_y(\pi/2) = \frac{1}{2}\begin{pmatrix}1 & 1\\ -1 & 1\end{pmatrix}, \quad \hat{K}_{-\pi/2}^{\text{Rot}} = \frac{1}{\sqrt{2}}R_y(-\pi/2) = \frac{1}{2}\begin{pmatrix}1 & -1\\ 1 & 1\end{pmatrix}. \tag{S5}$$

Note that for given $0 \le s \le 1$, $0 \le \kappa \le 1$, $0 \le \lambda \le 1$ (regardless of p), a quantum state

$$\hat{\gamma} = \begin{pmatrix}r & 0\\ 0 & 1-r\end{pmatrix}, \qquad r = \frac{s\lambda + (1-s)/2}{1+s(\lambda-\kappa)}. \tag{S6}$$

becomes a fixed point, satisfying $\mathcal{N}(\hat{\gamma}) = \hat{\gamma}$. Therefore, we choose such $\hat{\gamma}$ as a reference state to obtain the backward quantum channel

$$\tilde{\mathcal{N}}(\hat{\rho}) = \sum_x \hat{K}_x^{\mathcal{R}}\hat{\rho}\hat{K}_x^{\mathcal{R}\dagger}, \tag{S7}$$

where $\{\hat{K}_x\}$ is the Kraus operators describing the forward channel and $\hat{K}_x^{\mathcal{R}} = \hat{\gamma}^{1/2}\hat{K}_x^{\dagger}\hat{\gamma}^{-1/2}$.

We can also directly check that the backward channel is in fact the same as the forward channel, by directly comparing the two channels as

$$\tilde{\mathcal{N}}(\hat{\rho}) = \sum_x \hat{K}_x^{\mathcal{R}}\hat{\rho}\hat{K}_x^{\mathcal{R}\dagger} = \sum_x \hat{K}_x\hat{\rho}\hat{K}_x^{\dagger} = \mathcal{N}(\hat{\rho}). \tag{S8}$$

S1.2 Covariant and incovariant quantum channel

A quantum channel $\mathcal{N}_{\text{cov}}$ is called *covariant* if it satisfies the following symmetry condition with respect to the two group transformations $U(\theta)$ and $V(\theta)$,

$$\mathcal{N}_{\text{cov}}(U(\theta)\hat{\rho}U^{\dagger}(\theta)) = V(\theta)\mathcal{N}_{\text{cov}}(\hat{\rho})V^{\dagger}(\theta) \tag{S9}$$

for all θ. In this work, we focus on the covariance with respect to the stationary state $\hat{\gamma}$ by taking $U(\theta) = V(\theta) = U_{\hat{\gamma}}(\theta) = \hat{\gamma}^{-i\theta}$. In particular, when the stationary state $\hat{\gamma}$ is diagonal in the z-axis, $U_{\hat{\gamma}}(\theta)$ is essentially the rotation operator with respect to the z-axis. A covariant channel enjoys an important property that all the backward channel with rotation $U_{\hat{\gamma}}(\theta)$ yields the same dynamics, i.e.,

$$\tilde{\mathcal{N}}^{\theta}_{\text{cov}}(\hat{\rho}) = U^{\dagger}_{\hat{\gamma}}(\theta)\tilde{\mathcal{N}}_{\text{cov}}(U_{\hat{\gamma}}(\theta)\hat{\rho}U^{\dagger}_{\hat{\gamma}}(\theta))U_{\hat{\gamma}}(\theta) = \tilde{\mathcal{N}}_{\text{cov}}(\hat{\rho}) \qquad \forall\theta, \tag{S10}$$

where we denote

$$\tilde{\mathcal{N}}^{\theta}(\hat{\rho}) := U^{\dagger}_{\hat{\gamma}}(\theta)\tilde{\mathcal{N}}(U_{\hat{\gamma}}(\theta)\hat{\rho}U^{\dagger}_{\hat{\gamma}}(\theta))U_{\hat{\gamma}}(\theta) \tag{S11}$$

as the *rotated* Petz recovery map.

For the quantum channel $\mathcal{N}$ described in Eq. (S1), we note that taking the parameter $s = 1$ leads to a covariant channel, as it only contains dissipation $\mathcal{D}_{\kappa,\lambda}$. On the other hand, the channel is not covariant in general when $s \neq 1$ as the mixed rotation $\mathcal{R}_y^{\pi/2}$ gives rise to the transformation of off-diagonal elements, thus breaking the covariance. In this case, the different rotated Petz recovery maps induce different dynamics, i.e., $\tilde{\mathcal{N}}^{\theta}(\hat{\rho}) \neq \tilde{\mathcal{N}}^{\theta'}(\hat{\rho})$ for $\theta \neq \theta'$. Nevertheless, we

can always construct the corresponding covariant channel $\mathcal{N}_{\rm cov}$ for an incovariant channel $\mathcal{N}$ with $s \neq 1$ such that the covariant channel $\mathcal{N}_{\rm cov}$ gives the same dynamics as $\mathcal{N}$ for "diagonal" quantum states with respect to the eigenstates of the stationary state $\hat{\gamma}$. This can be done by taking the following parameters for the covariant channel ($s' = 1$) as

$$\kappa' = \left(\frac{1-s}{2}\right) + s\kappa, \quad \lambda' = \left(\frac{1-s}{2}\right) + s\lambda. \tag{S12}$$

We can easily check that

$$\mathcal{N}(\hat{\rho}_{\rm diag})|_{p,s,\kappa,\lambda} = \mathcal{N}(\hat{\rho}_{\rm diag})|_{p'=p,s'=1,\kappa',\lambda'}, \tag{S13}$$

for any diagonal state $\hat{\rho}_{\rm diag} = \begin{pmatrix} q & 0 \\ 0 & 1-q \end{pmatrix}$ with $0 \leq q \leq 1$. In this sense, $\mathcal{N}_{\rm cov} = \mathcal{N}|_{p'=p,s'=1,\kappa',\lambda'}$ can be considered as a covariant counterpart a given quantum channel $\mathcal{N}|_{p,s,\kappa,\lambda}$.

S2 Experimental details

S2.1 State preparation

In our experiment, the initial state $\hat{\rho}^I$ is a mixed state of the following form,

$$\hat{\rho}^I = p_0^I \left|\phi_0^I\right\rangle \left\langle\phi_0^I\right| + p_1^I \left|\phi_1^I\right\rangle \left\langle\phi_1^I\right|, \tag{S14}$$

with $p_0^I = 4/5, p_1^I = 1/5$ and $\left|\phi_0^I\right\rangle = \sin(\pi/6)\left|0\right\rangle - i\cos(\pi/6)\left|1\right\rangle, \left|\phi_1^I\right\rangle = \cos(\pi/6)\left|0\right\rangle + i\sin(\pi/6)\left|1\right\rangle$, encoded using the polarization degree of freedom ($\left|0\right\rangle$ for horizontal polarization and $\left|1\right\rangle$ vertical polarization) of photons. The state is prepared using a half-wave plate (HWP), several phase retarders (PRs), and a basis transformation realized by an HWP and a quarter-wave plate (QWP). The first HWP prepares the horizontally polarized single photon $\hat{\rho}_0^I = \left|0\right\rangle\left\langle 0\right|$ into $\hat{\rho}_1^I = (\sqrt{p_0^I}\left|0\right\rangle + \sqrt{p_1^I}\left|1\right\rangle)(\sqrt{p_0^I}\left\langle 0\right| + \sqrt{p_1^I}\left\langle 1\right|)$. The following PRs are equivalent to a phase damping channel as they erase all the off-diagonal terms of $\hat{\rho}_1^I$. Finally, the basis transformation $\hat{U} = \left|\phi_0^I\right\rangle\left\langle 0\right| + \left|\phi_1^I\right\rangle\left\langle 1\right|$ is applied to obtain the initial state $\hat{\rho}^I$. The evolved state $\hat{\rho}^F = \mathcal{N}(\hat{\rho}^I)$

is prepared in a similar way. Yet the reference state $\hat{\gamma}$ can be prepared in a simpler manner, as the state is diagonal so that the basis transformation operation is not required.

To verify that PRs correspond to a phase damping channel, we consider a single photon wave packet

$$|\psi\rangle = \int_t dt f(t)\,|t\rangle \otimes (\alpha\,|0\rangle + \beta\,|1\rangle), \tag{S15}$$

where $f(t)$ represents the amplitude of wave function in the time domain. When a PR is included, the vertically polarized component is retarded by n-wavelength compared to the horizontally polarized component. Here, n is determined by the length of the PR, fabricated from quartz crystal. Therefore, the wave function in the time domain is retarded by time τ, which leads to

$$|\psi(\tau)\rangle = \int_t dt f(t)\,|t\rangle \otimes \alpha\,|0\rangle + \int_t dt f(t+\tau)\,|t\rangle \otimes \beta\,|1\rangle). \tag{S16}$$

Consequently, the density matrix can be written as

$$|\psi(\tau)\rangle\,\langle\psi(\tau)| = \begin{pmatrix} |\alpha|^2 \int_{t_1}\int_{t_2} dt_1 dt_2 f(t_1) f^*(t_2)\,|t_1\rangle\,\langle t_2| & \alpha\beta^* \int_{t_1}\int_{t_2} dt_1 dt_2 f(t_1) f^*(t_2+\tau)\,|t_1\rangle\,\langle t_2| \\ \alpha^*\beta \int_{t_1}\int_{t_2} dt_1 dt_2 f(t_1+\tau) f^*(t_2)\,|t_1\rangle\,\langle t_2| & |\beta|^2 \int_{t_1}\int_{t_2} dt_1 dt_2 f(t_1+\tau) f^*(t_2+\tau)\,|t_1\rangle\,\langle t_2| \end{pmatrix}. \tag{S17}$$

By tracing out the time degree of the photon, the state becomes

$$\hat{\rho}_{\text{polarization}}(\tau) = \begin{pmatrix} |\alpha|^2 & \alpha\beta^* F(\tau) \\ \alpha^*\beta F(\tau) & |\beta|^2 \end{pmatrix}, \tag{S18}$$

where $F(\tau) = \int_t dt f(t) f^*(t+\tau)$. If $f(t)$ is a Gaussian function, then $F(\tau)$ also becomes a Gaussian function. In our experiment, we calibrate the value of $F(\tau)$ for the injected single photon to have the retarded number 180 with the photon wavelength λ_p, by using the polarization Mach-Zehnder interferometer *(67)*. This leads to $F(\tau = 180\lambda_p) = 0.3161$.

S2.2 Experimental realization of the quantum channels

Incovariant channel.—The incovariant channel in Eq. (S1) consists of three parts. The first part is the identity channel happened with probability p. The second is the coherence transfer part

$\mathcal{R}_y^{\pi/2}$, which accounts for $(1-p)(1-s)$ of the whole channel. The final part is the dissipation part $\mathcal{D}_{\kappa,\lambda}$. Instead of implementing the three parts individually, we find a simpler way to simultaneously realize the identity part and the rotation part by realizing a partial rotation channel $\mathcal{R}_y^{\varphi}(\hat{\rho}) = \cos\varphi\hat{\rho} + (1-\cos\varphi)\mathcal{R}_y^{\pi/2}(\hat{\rho})$.

In order to implement $\mathcal{R}_y^{\varphi}$, we first study the effect of $\mathcal{R}_y^{\varphi}$ on a qubit state. The channel shrinks the Bloch sphere equally along the $x-z$ plane. When $\phi = \pi/2$, all the points are projected onto the y axis and $\mathcal{R}_y^{\varphi}$ degenerates into $\mathcal{R}_y^{\pi/2}$. As the phase damping channel shrinks the Bloch sphere in a similar way along $x-y$ plane and can be easily realized with several PRs. Therefore, the channel $\mathcal{R}_y^{\varphi}$ can be realized by the following three steps: i) apply a coordinate transformation operation $R_x^{\pi/2} = e^{-i\frac{\pi}{2}\cdot\frac{\omega_x}{2}}$ on the Bloch sphere, ii) apply the phase damping channel, and iii) apply the reverse coordinate transformation $R_x^{\pi/2^{\dagger}}$. The coordinate transformation operation $R_x^{\pi/2}$ is implemented by a $45°$ oriented QWP in the experiment as well as its reverse transformation with a $-45°$ oriented QWP. The parameter φ in $\mathcal{R}_y^{\varphi}$ is related to the damping degree $F(\tau)$ ($\cos\varphi = F(\tau)$) of the phase damping channel, which is controlled by the number of retarded waves by the PRs. In our experiment, $\cos\varphi$ is calibrated to be 0.3161.

As for the dissipation part $\mathcal{D}_{\kappa,\lambda}$, we choose $\kappa = \lambda = 0$. So according to Eq. (S2), it deterministically dissipates any input state into the vertically polarized state. It is not a simple task to fulfill with linear photonic devices, so we only dissipate any input state into the vertically polarized state with a constant probability, which only causes a reduction of the proportion of dissipation part in the whole channel. This is achieved by first implementing the channel $\mathcal{R}_y^{\pi/2}$, which projects any state in the Bloch sphere onto the y axis, and then filtering the state by a vertically oriented polarizer with constant survival probability $\frac{1}{2}$ when ignoring transmission loss.

The rotation channel and dissipation channel together constitute the incovariant channel we studied. As shown in Fig. 1B in the main text, the transmitted part at the first $50:50$

non-polarizing beam splitter (NPBS) corresponds to the rotation channel, while the reflected part corresponds to the dissipation channel. Due to the limited success probability of $\mathcal{D}_{\kappa,\lambda}$ and transmission loss caused by optical components, the proportion of the channel $\mathcal{D}_{\kappa,\lambda}$ in $\mathcal{N}$ is calibrated to be 0.09393 at the end of the second NPBS. Using these parameters, the channel parameters p and s can be derived.

Covariant Channel.— By taking the quantum channel $\mathcal{N}$ with the parameters $s \neq 1$ and $\kappa = \lambda = 0$, its covariant counterpart $\mathcal{N}_{\text{cov}}$ can be determined by the parameters $s' = 1$ and $\kappa' = \lambda' = \frac{1-s}{2}$ (see Eq. (S12)), which deterministically dissipates any input state into the reference state $\hat{\gamma}$. Similarly, this channel can be realized with a constant survival probability. Moreover, we only need to prepare the state $\hat{\gamma}$ after the channel $\mathcal{D}_{\kappa,\lambda}$ is accomplished. The state preparation method is introduced in the previous subsection. In this manner, no matter the input state, the output state must be $\hat{\gamma}$.

The other difference of $\mathcal{N}_{\text{cov}}$ is that the coherence transfer part is not needed, so the transmitted part of the first NPBS corresponds to the identity channel. No further operations are needed for the transmitted photons apart from some attenuation, which is realized by a tunable attenuator, in order to set the proportion of transmitted photons equal to $p = 0.2864$ at the end of the second NPBS. As for the corresponding backward channel, it is exactly the forward channel in the covariant case.

S2.3 The correspondence between the circuit model and its optical implementation

For an arbitrary single-qubit quantum channel, two ancillary qubits are needed for the channel construction by using the Stinespring dilation. By introducing a joint unitary operation of the target and ancillary qubits and tracing out the ancillary qubits, we can get the evolution of the channel. However, using single-qubit unitary operations and CNOT gates to decompose

the dilated unitary operation will entail significant experimental complexity and considerable accumulation of noise. The key to achieving a higher channel precision lies in simplifying the circuit design and reducing the number of operations. One possible way is to introduce more ancillary qubits or using even larger Hilbert space *(68)*, which is particularly suitable for photons due to their rich degrees of freedoms (DOFs). In this work, we encode the qubits using four photonic DOFs: temporal (q_0), path (q_1), polarization (q_2) and another path (q_3) with q_2 as the target qubit and q_3 is used for the measurement-controlled gate. The schematic for the circuit to implement incovariant and covariant channels are depicted in Fig. S1, respectively, which is greatly simplified by involving multiple DOFs using simple optical elements.

The temporal DOF is deliberately chosen as the ancillary mode. The temporal DOF is normally robust against noise in optical experiments, since two temporal modes in a common path often experience the same noise and effect on their relative delay cancel out. When coupled with polarization and path DOFs, the continuous nature of the temporal DOF and its larger Hilbert space enable the implementation of the Toffoli gate and control-control-$\mathcal{R}_x(\theta)$ gates with two controlled temporal delays. These delays are readily achieved using phase retarders with different thicknesses (denoted as PR1 and PR2) on a particular path in optical experiment, thus enhancing the gate fidelity.

The experimental implementation of the incovariant channel are also simplified. The partial rotation channel $\mathcal{R}^{\varphi}$ is achieved with temporal DOF using a simple three-element QWP-PR-QWP sandwich structure, benefiting from easier experimental implementation and thus minimizing errors and improving precision. For the implementation of the channel $\mathcal{D}(\hat{\rho})$, we can couple the polarization with another ancillary mode q_3 using a polarizing beam splitter (PBS) and conditionally flip the polarization qubit based on the measurement outcome on q_3. If the measurement outcome is 0, i.e. the photon is transmitted by the PBS, we apply the identity operation which will leave the polarization qubit unchanged. If the measurement outcome is 1,

i.e. the photon is reflected by the PBS, we apply the X gate to flip the polarization qubit. In our experiment, we did not fully implement the measurement-controlled gate. Instead, we only focus on the case when the measurement result is 0 by using a polarizer in place of the PBS and discard the case when the measurement outcome is 1.

In addition to the higher precision of the channel construction, the precision of the state preparation and the measurement part are essential for the experiment. In optical experiments, PBS and HWP combination are normally used for parameter control. Instead, we utilize a rotating polarizer with superior extinction compared with a PBS, eliminating the need for a HWP and thereby simplifying the experimental design and reducing systematic error through decreased error accumulation *(69)*.

S2.4 Two-point measurements and its experimental realization

In traditional two-point projective measurement protocol, projective measurements are performed on the initial and final state, which destroy all the quantum coherence in the quantum states. To keep coherence terms in the quantum states, a two-point generalized measurement protocol was proposed in Ref. *(41)*. From the probability distribution of two-point measurement outcomes, the TPM quasi-probability distribution can be reconstructed. In the qubit case, we construct the initial and final measurements as follows:

$$\begin{aligned} \hat{M}_m &= \hat{M}_{(\mu,r)} = \hat{L}_r \hat{\Phi}^I_\mu \\ \hat{M}'_{m'} &= \hat{M}'_{(\nu,s)} = \hat{\Phi}^F_\nu \hat{L}_s, \end{aligned} \tag{S19}$$

where we define $\hat{L}_1 = \frac{\hat{\Pi}_0}{\sqrt{2}}$, $\hat{L}_2 = \frac{\hat{\Pi}_1}{\sqrt{2}}$, $\hat{L}_3 = \frac{\mathbb{1}}{2}$, and $\hat{L}_4 = \frac{\hat{S}}{2}$ with $\hat{S} = |0\rangle\langle 0| + i\,|1\rangle\langle 1|$, $\hat{\Pi}_i = |i\rangle\langle i|$, and $\hat{\Phi}^{I(F)}_{\mu(\nu)} = \left|\phi^{I(F)}_{\mu(\nu)}\right\rangle\left\langle\phi^{I(F)}_{\mu(\nu)}\right|$. Denominators in $\hat{L}_r$ are chosen to meet the completeness condition $\sum_m \hat{M}^\dagger_m \hat{M}_m = \mathbb{1}$ and $\sum_{m'} \hat{M}'^\dagger_{m'} \hat{M}'_{m'} = \mathbb{1}$. As $\hat{\Phi}^I_\mu$ and $\hat{\Phi}^F_\nu$ are projectors onto two different eigenstates $\mu = \{0,1\}$ and $\nu = \{0,1\}$, respectively, each measurement contains $2 \times 4 = 8$ effects. The probability distribution of two-point measurement outcomes (m, m') is

then written as

$$P_{\rightarrow}(m,m') = \mathrm{Tr}[\hat{M'}_{m'}\mathcal{N}(\hat{M}_m\hat{\rho}\hat{M}_m^{\dagger})\hat{M}'^{\dagger}_{m'}] = \mathrm{Tr}[\hat{M'}_{(\nu,s)}\mathcal{N}(\hat{M}_{(\mu,r)}\hat{\rho}\hat{M}^{\dagger}_{(\mu,r)})\hat{M}'^{\dagger}_{(\nu,s)}], \quad \text{(S20)}$$

To implement the protocol, the most important step is to realize the following two measurement operators $\{\hat{M}_m\} = \{\hat{M}_{(\mu,r)}\}$ and $\{\hat{M}'_{m'}\} = \{\hat{M}'_{(\nu,s)}\}$ before and after the quantum channel. According to the Naimark theorem *(70)*, generally, we need to couple our system qubit with an ancilla to realize an arbitrary operator. Therefore, two ancilla qubits and several two-qubit gates are needed to faithfully carry out the two-point measurement protocol, which is rather complicated and inefficient. To this end, we find out a much simpler way to individually realize each effect of the measurements through two successive "projection" methods. For instance, the measurement operator $\hat{M}_{(\mu,1)}$ and $\hat{M}_{(\mu,2)}$ can be realized by first projecting the initial state onto $|\phi^I_\mu\rangle$, and then projecting the resulting state onto the reference basis state $|0\rangle$ or $|1\rangle$. The coefficient $\frac{1}{\sqrt{2}}$ is neglected in this procedure and will be considered in the normalization stage (see below). When it comes to $\hat{M}_{(\mu,3)}$, we do not need to perform any extra operation after the first projection $\hat{\Phi}^I_\mu$ as $\hat{L}_3$ is proportional to the identity operation. For $\hat{M}_{(\mu,4)}$, we only need to apply the $\frac{\pi}{2}$-phase gate operation to realize $\hat{S}$ after the projection onto $\hat{\Phi}^I_\mu$. Eventually, all the effects in $\{\hat{M}_m\}$ can be implemented in the experiments, and the second measurement $\{\hat{M}'_{m'}\}$ can be realized in a similar manner.

As a combination, the two-point generalized measurement protocol totally gives rise to 64 measurement outcomes. To equivalently derive the probability distribution of the measurement outcomes, we individually realize the 64 combinations of the measurement effects and record the detected photon numbers for each experimental setting. After normalization of the registered 64 photon counts (some are divided by 2 or 4 according to their proportions determined by the measurement operators), the probability distribution of the two-point measurement outcomes is estimated.

In our experiment, the first projective measurement $\hat{\Phi}^I_\mu$ and $\hat{\Phi}^F_\nu$ is realized with a QWP and a polarizer. As for the second "projective" operation, it was realized by another polarizer followed by a half-cutted QWP. When operating on the projection of state $|0\rangle$ and $|1\rangle$, the QWP is rotated to the empty half, so the state does not go through it. When operating on the $\hat{M}_{(\mu,3)}$ and $\hat{M}'_{(\nu,3)}$ settings, the angle of the second polarizer is set at the same angle as the first one, and the QWP is also rotated to the empty half, which equivalently realizes the identity operation but shares the same transmission loss with other settings. When operating on the $\hat{M}_{(\mu,4)}$ and $\hat{M}'_{(\nu,4)}$ settings, the second polarizer is also set at the same angle as the first one, but the QWP is rotated to the nonempty half, serving as the $\frac{\pi}{2}$-phase gate. Similarly, the transmission loss of this setting is almost the same as other settings, due to the high transmittance of the QWP.

S2.5 Details of the quantum tomography techniques used in our experiments

Quantum process tomography.—Following the standard quantum process tomography (QPT) procedure *(71)*, we prepared a set informationally complete states $\{|\psi\rangle\} = \{|0\rangle, |1\rangle, |D\rangle = \frac{1}{\sqrt{2}}(|0\rangle + |1\rangle), |R\rangle = \frac{1}{\sqrt{2}}(|0\rangle + i|1\rangle)\}$ and input them into the channel. Then projective measurements of the mutually unbiased bases $\{|\psi'\rangle\langle\psi'|\}$ ($\{|\psi'\rangle\} = \{|0'\rangle, |1'\rangle, |D'\rangle = \frac{1}{\sqrt{2}}(|0'\rangle + |1'\rangle), |A'\rangle = \frac{1}{\sqrt{2}}(|0'\rangle - |1'\rangle), |R'\rangle = \frac{1}{\sqrt{2}}(|0'\rangle + i|1'\rangle), |L'\rangle = \frac{1}{\sqrt{2}}(|0'\rangle - i|1'\rangle)\}$) are performed on each evolved state $\varepsilon(|\psi\rangle\langle\psi|)$ and the full QPT data $\{\langle\psi'|\varepsilon(|\psi\rangle\langle\psi|)|\psi'\rangle\}$ are obtained. Finally, by using the minimum standard derivation method, the chi matrix χ_{tomo} of an unknown qubit channel is estimated. In our paper, the fidelity of two quantum channel is defined by the overlap of their chi matrix, that is $F = \mathrm{Tr}(\sqrt{\chi_{tomo}}\chi\sqrt{\chi_{tomo}})/(\mathrm{Tr}(\chi_{tomo})\,\mathrm{Tr}(\chi))$.

Reconstructing entropy production.—Since the entropy production ω is not a quantum observable, we can not directly measure it in experiments. However, from the definition of entropy production, it can be reconstructed from other measurable quantities. According to the defini-

tion $\omega_{ij\to kl}^{\mu\to\nu} = \delta s^{\mu\to\nu} - \delta q_{ij\to kl}$, once the single-shot entropy difference $\delta s^{\mu\to\nu}$ and information exchange $\delta q_{ij\to kl}$ are calculated, the entropy production is obtained. To derive $\delta s^{\mu\to\nu}$, we apply eigenvalue decomposition on the reconstructed quantum states $\hat{\rho}^I$ and $\hat{\rho}^F = \mathcal{N}(\hat{\rho}^I)$, from which, $\{p_\mu^I\}$ and $\{p_\nu^F\}$ are acquired and the single-shot entropy difference is calculated. By definition, the information exchange is the logarithm of the ratio of forward transition probability $T_{ij\to kl}$ to backward transition probability $\tilde{T}^*_{ij\leftarrow kl}$, which is solely determined by the quantum channel. Therefore, the full QPT data $\{\langle\psi'|\,\varepsilon(|\psi\rangle\langle\psi|)\,|\psi'\rangle\}$ is enough to reconstruct the transition probabilities and consequently the information exchange. Take the forward channel as an example, we have listed several cases below,

$$T_{00\to1'1'} = \langle 1'|\,\mathcal{N}(|0\rangle\langle 0|)\,|1'\rangle\,, \tag{S21}$$

$$T_{11\to0'0'} = \langle 0'|\,\mathcal{N}(|1\rangle\langle 1|)\,|0'\rangle\,, \tag{S22}$$

$$T_{01\to0'0'} = \langle 0'|\left[\mathcal{N}(|D\rangle\langle D|) + i\mathcal{N}(|R\rangle\langle R|) - \frac{1+i}{2}\mathcal{N}(|0\rangle\langle 0|) - \frac{1+i}{2}\mathcal{N}(|1\rangle\langle 1|)\right]|0'\rangle\,, \tag{S23}$$

$$T_{10\to1'1'} = \langle 1'|\left[\mathcal{N}(|D\rangle\langle D|) - i\mathcal{N}(|R\rangle\langle R|) - \frac{1-i}{2}\mathcal{N}(|0\rangle\langle 0|) - \frac{1-i}{2}\mathcal{N}(|1\rangle\langle 1|)\right]|1'\rangle\,, \tag{S24}$$

$$\begin{aligned} T_{00\to0'1'} = &\frac{1}{2}[\langle D'|\,\mathcal{N}(|0\rangle\langle 0|)\,|D'\rangle - \langle A'|\,\mathcal{N}(|0\rangle\langle 0|)\,|A'\rangle] \\ &- \frac{i}{2}[\langle R'|\,\mathcal{N}(|0\rangle\langle 0|)\,|R'\rangle - \langle L'|\,\mathcal{N}(|0\rangle\langle 0|)\,|L'\rangle], \end{aligned} \tag{S25}$$

$$T_{01\to1'0'} = \frac{1}{2}\langle D'|\left[\mathcal{N}(|D\rangle\langle D|) + i\mathcal{N}(|R\rangle\langle R|) - \frac{1+i}{2}\mathcal{N}(|0\rangle\langle 0|) - \frac{1+i}{2}\mathcal{N}(|1\rangle\langle 1|)\right]|D'\rangle \tag{S26}$$

$$\begin{aligned} &- \frac{1}{2}\langle A'|\left[\mathcal{N}(|D\rangle\langle D|) + i\mathcal{N}(|R\rangle\langle R|) - \frac{1+i}{2}\mathcal{N}(|0\rangle\langle 0|) - \frac{1+i}{2}\mathcal{N}(|1\rangle\langle 1|)\right]|A'\rangle \\ &+ \frac{i}{2}\langle R'|\left[\mathcal{N}(|D\rangle\langle D|) + i\mathcal{N}(|R\rangle\langle R|) - \frac{1+i}{2}\mathcal{N}(|0\rangle\langle 0|) - \frac{1+i}{2}\mathcal{N}(|1\rangle\langle 1|)\right]|R'\rangle \\ &- \frac{i}{2}\langle L'|\left[\mathcal{N}(|D\rangle\langle D|) + i\mathcal{N}(|R\rangle\langle R|) - \frac{1+i}{2}\mathcal{N}(|0\rangle\langle 0|) - \frac{1+i}{2}\mathcal{N}(|1\rangle\langle 1|)\right]|L'\rangle\,. \end{aligned} \tag{S27}$$

S2.6 Experimental results of the incovariant channel

The parameter are calibrated as $p = 0.2864$ and $s = 0.1316$. For incovariant channel, the two-point measurement outcome can be found in Fig. S2, which contains the transitions between the eigenstates of the input state and output state. From which the quasi-probability can be reconstructed, and the real part and imaginary part of the quasi-probability can be found in Fig. S3 and Fig. S4 respectively. We can see that the imaginary part of the quasi-probability equals to zero for the forward channel and the reverse channel when $\theta = 0$, when the rotated angle does not equal to zero $\theta \neq 0$, the non-zero imaginary part of the quasi-probability can be observed in the experiment. We also found that for incovariant channel, the distance between experimental quasi-probabilities and theory one are close to the zero, which show a good aggrement with theory prediction (see Table. S1). For three different rotated reversal channel, we test the QFT for both log-magnitude and phase of the ratio of the quasi-probabilities $P_{\rightarrow}(\omega)/P^{\theta}_{\leftarrow}(-\omega^{*})$ (see Fig. S5), the plane are the theoretical predictions of the log-magnitude and the phase of the quasi-probability ratio for different rotated angle θ, the experimental data are all in the theoretical plane.

S2.7 Experimental results of the covariant channel

Probability distribution of the two-point measurement outcomes for the channel $\mathcal{N}_{\text{cov}}$ and its corresponding backward channels are shown in Fig. S6, from which the TPM quasi-probability distributions are calculated, whose real and imaginary parts are presented in Fig. S7 and Fig. S8. The experimental results agree well with the theoretical predictions. Negative values of the quasi-probabilities are obviously observed. However, most of the imaginary parts of TPM quasi-probabilities are smaller than their error bars. Some violations are caused by experimental errors. For covariant channel, the distance between experimental quasi-probabilities and theory one are close to the zero, which show a good aggrement with theory prediction (see Table. S1).

The generalized quantum Crooks FT is also validated in the covariant channels, as shown in Fig. S9. Similarly, negative entropy productions are observed, but imaginary entropy productions vanished even in the rotated Petz recovery map. Compared to the experimental results in the main text, it is demonstrated that the lack of covariant property of the quantum channel is vital to the imaginary part of entropy production and quasi-probability.

S2.8 Transition probability and its fourier transform

It has been shown in the above section that the imaginary entropy production vanishes in the covariant channel, which indicates the broken symmetry of the quantum channel. In this section, we experimentally show that the imaginary entropy production also provides additional information about how it responds to the transformation $\hat{\gamma}^{i\theta}$ in the frequency domain. As seen in the Fig. S10, transition probabilities between the rotated eigenstates $\hat{\gamma}^{i\theta}\left|\phi_0^I\right\rangle$ and $\hat{\gamma}^{i\theta}\left|\phi_0^F\right\rangle$ are fitted with a cosine function. The fast Fourier transformation (FFT) of the experimentally derived discrete transition probabilities is given in Fig. S11. The peaks are located at $\omega = 0$ and $\omega = 0.2647$, which exactly match the theoretical predictions of ω_I (0 and $\pm\log(\frac{r}{1-r})$).

S2.9 The relation between the negativity of quasi-probability and the coherence of the input state.

The channel remains the same, we can get different negativity values of quasi-probability by changing the coherence of the input state. The input state is $\hat{\rho}^I = \sum_\mu p_\mu^I \left|\phi_\mu^I\right\rangle\left\langle\phi_\mu^I\right|$ with eigenvalues $p_0^I = 4/5$ and $p_1^I = 1/5$, and the eigenstates is $\left|\phi_0^I\right\rangle = \sin(\alpha)\left|0\right\rangle - i\cos(\alpha)\left|1\right\rangle$, $\left|\phi_1^I\right\rangle = \cos(\alpha)\left|0\right\rangle + i\sin(\alpha)\left|1\right\rangle$, we can change eigenstates from $\alpha = \pi/6$ to $\alpha = \pi/4$. As seen in the Fig. S12, when $\alpha = \pi/4$, the absolute negativity value of quasi-probability is bigger than $\alpha = \pi/6$.

S3 Theoretical derivation

S3.1 Entropy production

By introducing the complex-valued transition amplitude $T_{ij\to kl}^{\mu\to\nu}$, we can reconstruct the average entropy production $\overline{\omega}$ in terms of the transition amplitude $P_{\to}(\omega)$ with complexed-valued stochastic entropy production $\omega_{ij\to kl}^{\mu\to\nu}$ for each transition. The form of the average entropy production can be written as $\overline{\omega} = \sum_{\omega} \omega P_{\to}(\omega) = S(\hat{\rho}^I \| \hat{\gamma}) - S(\hat{\rho}^F \| \hat{\gamma}) = \langle \omega_R \rangle + i \langle \omega_I \rangle$, where $\langle \omega_R \rangle$ and $\langle \omega_I \rangle$ are the real and imaginary part of the average entropy production respectively. By averaging over all possible transitions for the real part of the entropy production, we have

$$
\begin{aligned}
\langle \omega_R \rangle &= \text{Real}\left[\sum_{\omega} \omega P_{\to}(\omega)\right] \\
&= \sum_{\mu,\nu,i,j,k,l} p_\mu^I T_{ij\to kl}^{\mu\to\nu} \ln\left(\frac{p_\mu^I \sqrt{r_k r_l}}{p_\nu^F \sqrt{r_i r_j}}\right) \\
&= \sum_{\mu,\nu,i,j,k,l} p_\mu^I T_{ij\to kl}^{\mu\to\nu} \Big[\ln p_\mu^I - \ln p_\nu^F \\
&\quad + \frac{\ln r_k + \ln r_l}{2} - \frac{\ln r_i + \ln r_j}{2}\Big].
\end{aligned} \tag{S28}
$$

From the completeness relation we note that

$$
\begin{aligned}
&\sum_{\nu,i,j,k,l} p_\mu^I T_{ij\to kl}^{\mu\to\nu} = p_\mu^I, \\
&\sum_{\mu,i,j,k,l} p_\mu^I T_{ij\to kl}^{\mu\to\nu} = p_\nu^F, \\
&\sum_{\mu,\nu,j,k,l} p_\mu^I T_{ij\to kl}^{\mu\to\nu} = \langle i| \hat{\rho}^I |i\rangle, \\
&\sum_{\mu,\nu,i,k,l} p_\mu^I T_{ij\to kl}^{\mu\to\nu} = \langle j| \hat{\rho}^I |j\rangle, \\
&\sum_{\mu,\nu,i,j,l} p_\mu^I T_{ij\to kl}^{\mu\to\nu} = \langle k| \hat{\rho}^F |k\rangle, \\
&\sum_{\mu,\nu,i,j,k} p_\mu^I T_{ij\to kl}^{\mu\to\nu} = \langle l| \hat{\rho}^F |l\rangle.
\end{aligned} \tag{S29}
$$

From this, we can rewrite the real part of the average entropy production as

$$\begin{aligned}\langle\omega_R\rangle &= \sum_\mu p^I_\mu \ln p^I_\mu - \sum_\nu p^F_\nu \ln p^F_\nu \\ &+ \frac{\sum_k \langle k|\,\hat\rho^F\,|k\rangle\, r_k \ln r_k + \sum_l \langle l|\,\hat\rho^F\,|l\rangle \ln r_l}{2} \\ &- \frac{\sum_i \langle i|\,\hat\rho^I\,|i\rangle \ln r_i + \sum_j \langle j|\,\hat\rho^I\,|j\rangle \ln r_j}{2}.\end{aligned} \tag{S30}$$

The first two terms correspond to $-S(\hat\rho^I) = \mathrm{Tr}[\hat\rho^I \ln \hat\rho^I]$ and $S(\hat\rho^F) = -\mathrm{Tr}[\hat\rho^F \ln \hat\rho^F]$, respectively. The remaining terms can be evaluated as

$$\begin{aligned}\sum_i \langle i|\,\hat\rho^I\,|i\rangle \ln r_i &= \sum_i \mathrm{Tr}[\hat\Pi_i \hat\rho^I \hat\Pi_i \ln r_i] \\ &= \mathrm{Tr}\left[\hat\rho^I \sum_i \left(\hat\Pi_i \ln r_i \hat\Pi_i\right)\right] \\ &= \mathrm{Tr}\left[\hat\rho^I \ln\left(\sum_i \hat\Pi_i r_i \hat\Pi_i\right)\right] \\ &= \mathrm{Tr}\left[\hat\rho^I \log\hat\gamma\right],\end{aligned} \tag{S31}$$

where we used $\hat\gamma = \sum_i r_i \hat\Pi_i = \sum_i \hat\Pi_i r_i \hat\Pi_i$. Similarly, we can express the other terms using $\hat\rho^I$, $\hat\rho^F$ and $\hat\gamma$. This leads to

$$\begin{aligned}\langle\omega_R\rangle &= \mathrm{Tr}[\hat\rho^I \ln\hat\rho^I] - \mathrm{Tr}[\hat\rho^F \ln\hat\rho^F] + \mathrm{Tr}[\hat\rho^F \ln\hat\gamma] \\ &- \mathrm{Tr}[\hat\rho^I \ln\hat\gamma] \\ &= S(\hat\rho^I\|\hat\gamma) - S(\hat\rho^F\|\hat\gamma).\end{aligned} \tag{S32}$$

For the imaginary part of the average entropy production, we have

$$\begin{aligned}
\langle \omega_I \rangle &= \text{Imag}\left[\sum_{\omega} \omega P_{\rightarrow}(\omega)\right] \\
&= \sum_{\mu,\nu,i,j,k,l} p_{\mu}^{I} T_{ij\rightarrow kl}^{\mu\rightarrow\nu} \ln\left(\frac{\sqrt{r_j r_l}}{\sqrt{r_i r_k}}\right) \\
&= \sum_{\mu,\nu,i,j,k,l} P_{i,j,k,l}^{\mu,\nu} \left[\frac{\ln r_j - \ln r_i}{2} - \frac{\ln r_l - \ln r_k}{2}\right] \\
&= \frac{\sum_j \langle j| \hat{\rho}^I |j\rangle \ln r_j - \sum_i \langle i| \hat{\rho}^I |i\rangle \ln r_i}{2} \\
&- \frac{\sum_l \langle l| \hat{\rho}^F |l\rangle \ln r_l - \sum_k \langle k| \hat{\rho}^F |k\rangle \ln r_k}{2} \\
&= \frac{\text{Tr}[\hat{\rho}^I \ln \hat{\gamma}] - \text{Tr}[\hat{\rho}^I \ln \hat{\gamma}]}{2} - \frac{\text{Tr}[\hat{\rho}^F \ln \hat{\gamma}] - \text{Tr}[\hat{\rho}^F \ln \hat{\gamma}]}{2} \\
&= 0,
\end{aligned} \tag{S33}$$

which completes the proof that $\overline{\omega} = \sum_{\omega} \omega P_{\rightarrow}(\omega) = S(\hat{\rho}^I \| \hat{\gamma}) - S(\hat{\rho}^F \| \hat{\gamma}) = \langle \omega_R \rangle + i \langle \omega_I \rangle$.

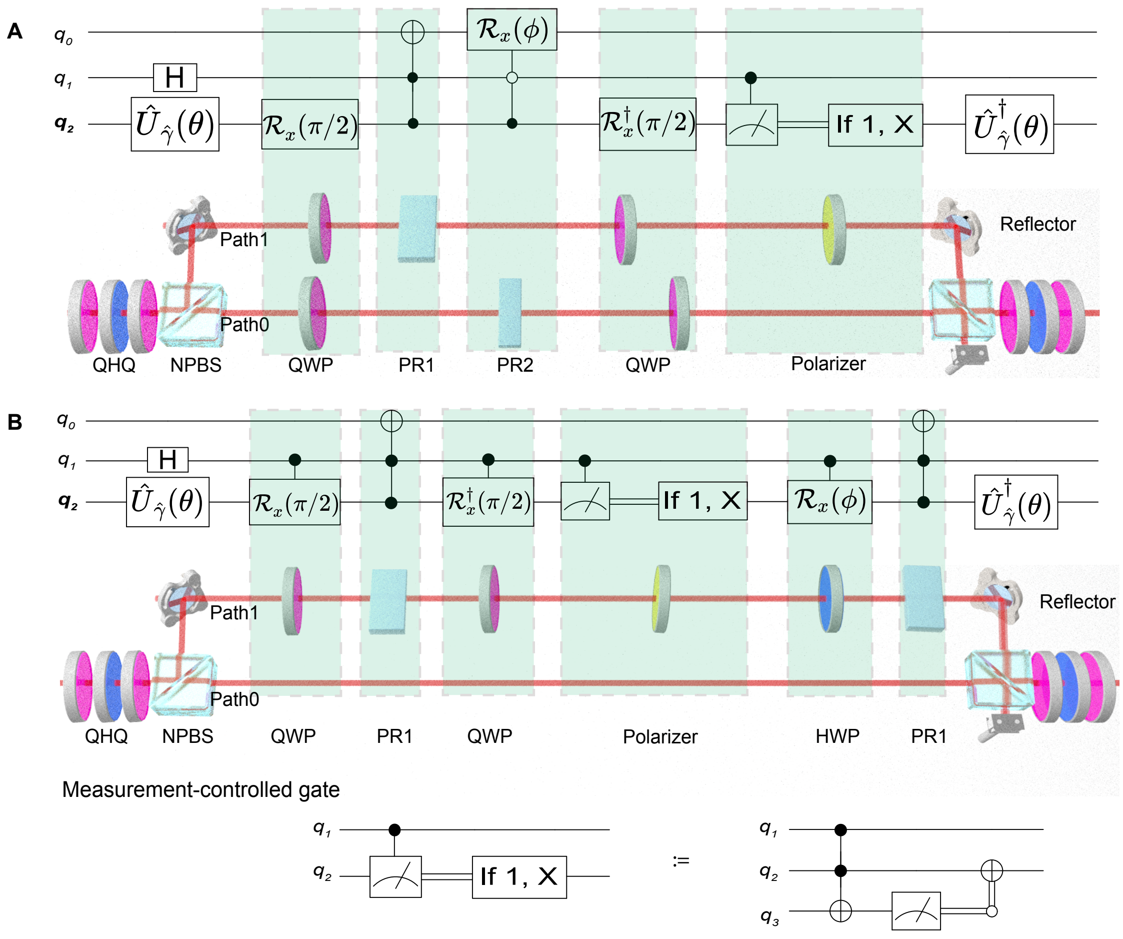

Fig. S1. The correspondence between the circuit model and the optical experiment for the incovariant channel (A) and covariant channel (B). The channel are encoded into four qubits: q_0, temporal mode, q_1, path mode, q_2, polarization mode, q_3, another path mode with q_2 as the target qubit and q_3 is used for the measurement-measurement part. The green dashed boxes show the correspondence between the circuit model and its optical implementation. H in the box of the circuit means the Hadamard operation, which is achieved by a non-polarizing beam splitter (NPBS) and introduce the ancillary mode q_1. $\hat{U}_{\hat{\gamma}}(\theta) = e^{-i\theta \ln \hat{\gamma}}$ is a unitary operation on the system qubit, which can be achieved by a QHQ combination (Q, quarter-wave plate, H, half-wave plates). $\mathcal{R}_x(\pi/2)$ and $\mathcal{R}_x(\phi)$ are the rotation of angle $\pi/2$ and ϕ around the x-axis of the Bloch sphere, respectively. PR1 and PR2 are phase retarders with different thicknesses. The measurement-controlled gate is achieved by introducing q_3 as the ancillary qubit, if the measurement outcome is 0, then apply identity operation and leave the polarization unchanged, if the measurement outcome is 1, then apply the X gate to flip the p olarization. In our experiment, we did not fully implement the measurement-controlled gate. Instead, we only focus on the case when the measurement result is 0 by using a polarizer in place of the PBS and discard the case when the measurement outcome is 1.

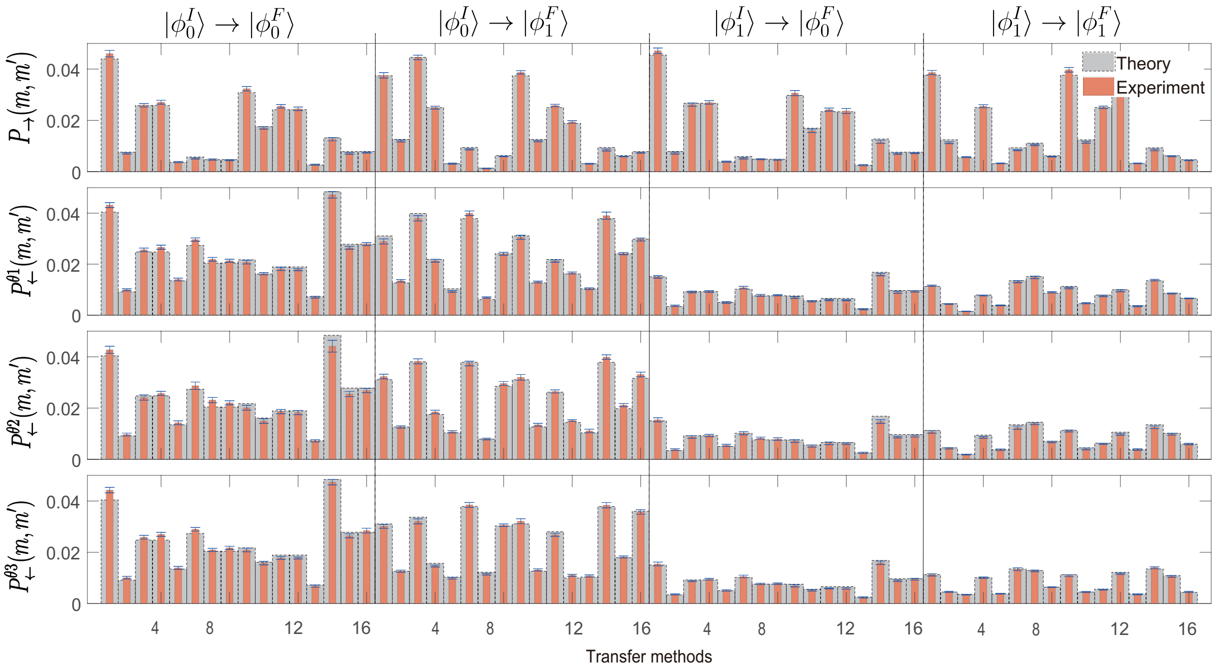

Fig. S2. Experimental results of the two-point measurement outcome probabilities for the forward channel $\mathcal{N}$ and the three different rotated Petz recovery channels $\tilde{\mathcal{N}}^{\theta}$. The shaded gray square stands for the theory value and the orange square stands for the experimental data of the two-point measurement outcome. Experimental data are plotted with error bars.

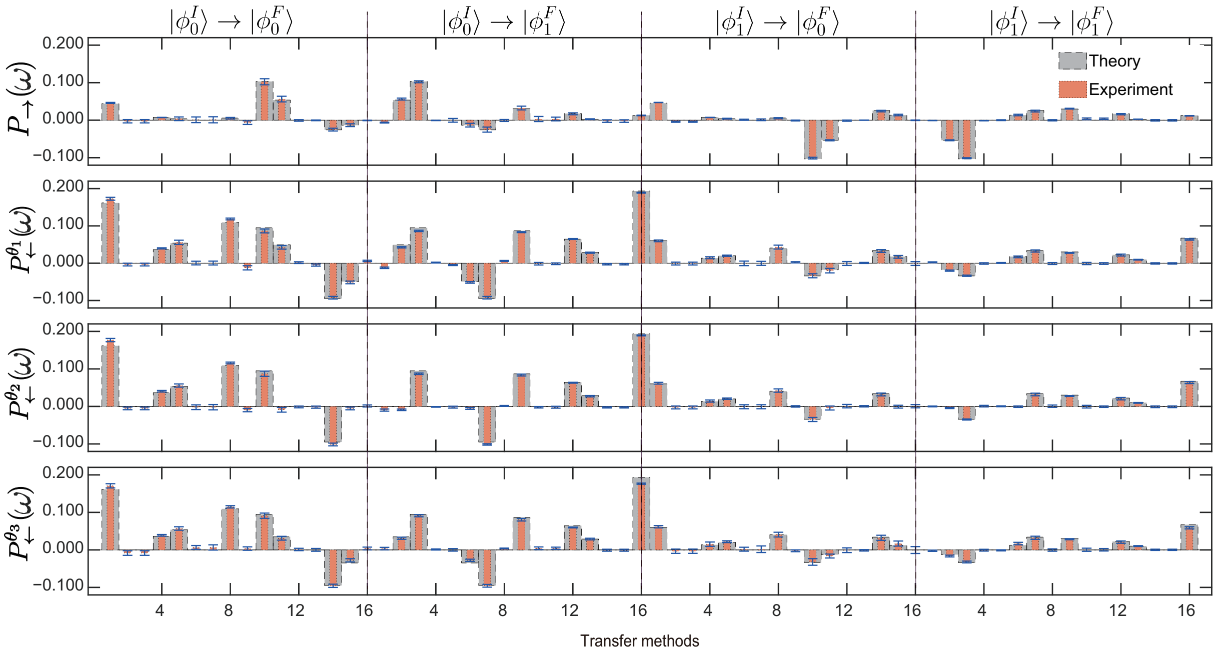

Fig. S3. Real parts of the TPM quasi-probability distributions of the incovariant channel. The shaded gray square stands for the theory value and the orange square stands for the experimental data of the quasi-probability distribution. Experimental data are plotted with error bars.

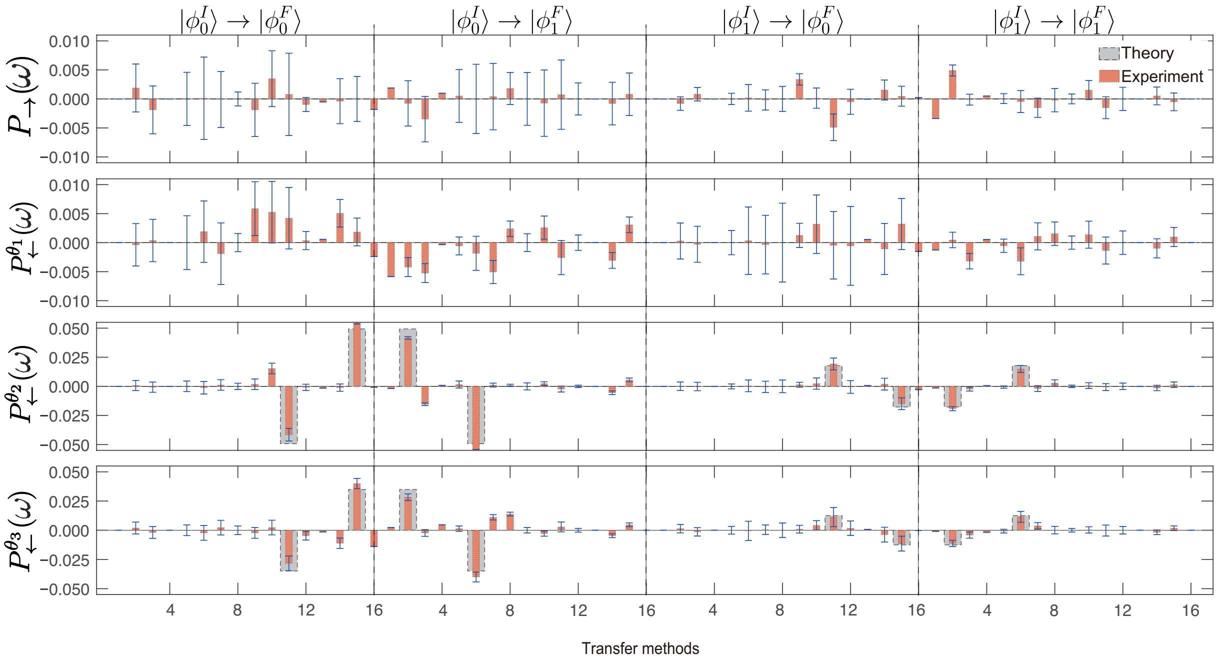

Fig. S4. Imaginary parts of the TPM quasi-probability distributions of the incovariant channel. The shaded gray square stands for the theory value and the orange square stands for the experimental data of the quasi-probability distribution. Experimental data are plotted with error bars.

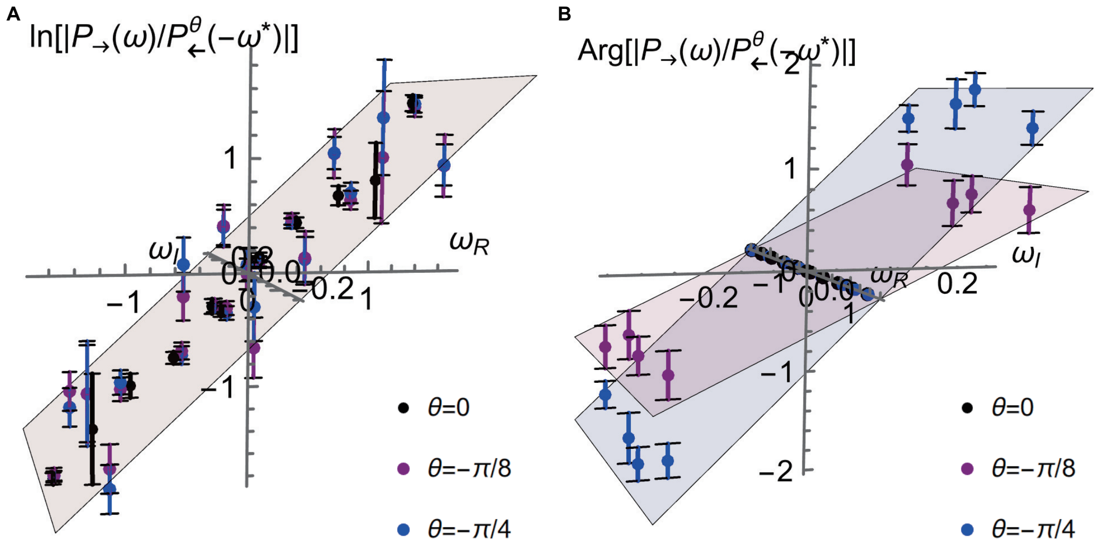

Fig. S5. Verification of the QFT. (A), Testing the QFT for the log-magnitude of the quasi-probability ratio $P_{\rightarrow}(\omega)/P^{\theta}_{\leftarrow}(-\omega^{*})$ with $\theta = 0$ (black), $-\pi/8$ (purple), and $-\pi/4$ (blue). The purple plane is the theoretical predictions for the log-magnitude of the quasi-probability ratio. **(B)**,Testing the QFT for the phase of $P_{\rightarrow}(\omega)/P^{\theta}_{\leftarrow}(-\omega^{*})$ with $\theta = 0$ (black), $-\pi/8$ (purple), and $-\pi/4$ (blue). The purple and blue plane are the theoretical predictions for the phase of the quasi-probability ratio for $\theta = -\pi/8$ and $\theta = -\pi/4$ respectively. Experimental data are plotted with error bars.

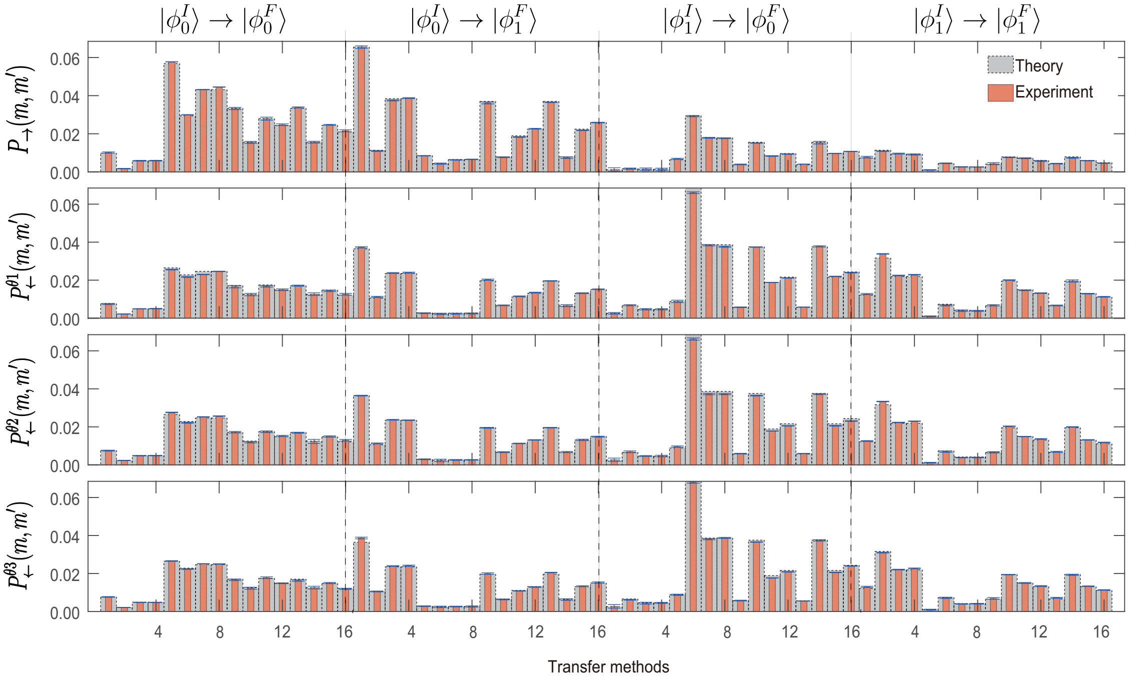

Fig. S6. Experimental results of the two-point measurement outcomes for the forward channel $\mathcal{N}_{\rm cov}$ and the three different rotated Petz recovery channels of $\mathcal{N}_{\rm cov}$. The shaded gray square stands for the theory value and the orange square stands for the experimental data of the quasi-probability distribution. Experimental data are plotted with error bars.

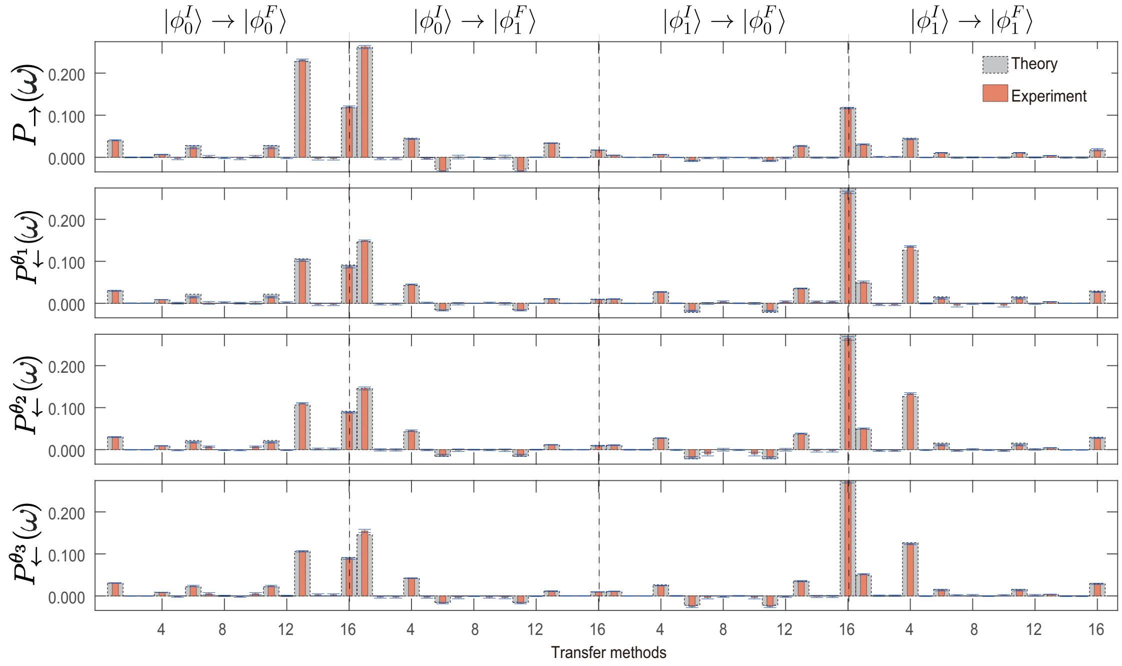

Fig. S7. Real parts of the TPM quasi-probability distributions of the covariant channel. The shaded gray square stands for the theory value and the orange square stands for the experimental data of the quasi-probability distribution. Experimental data are plotted with error bars.

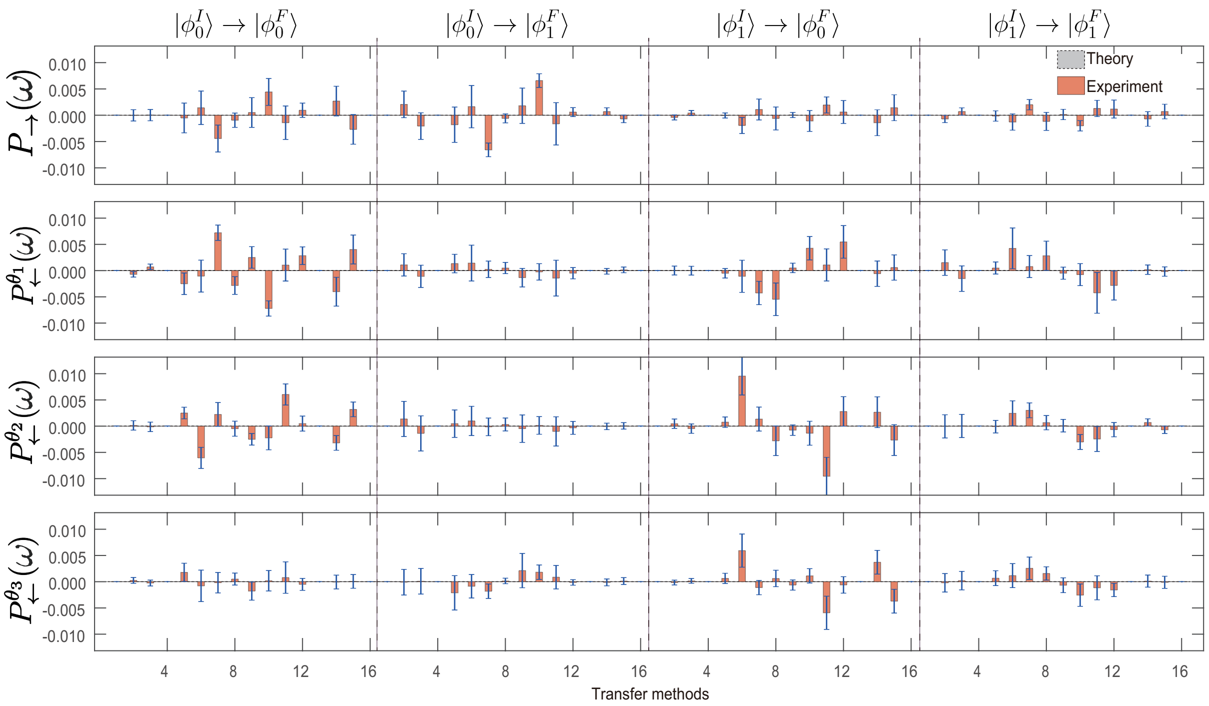

Fig. S8. Imaginary parts of the TPM quasi-probability distributions of the covariant channel. The shaded gray square stands for the theory value and the orange square stands for the experimental data of the quasi-probability distribution. Experimental data are plotted with error bars.

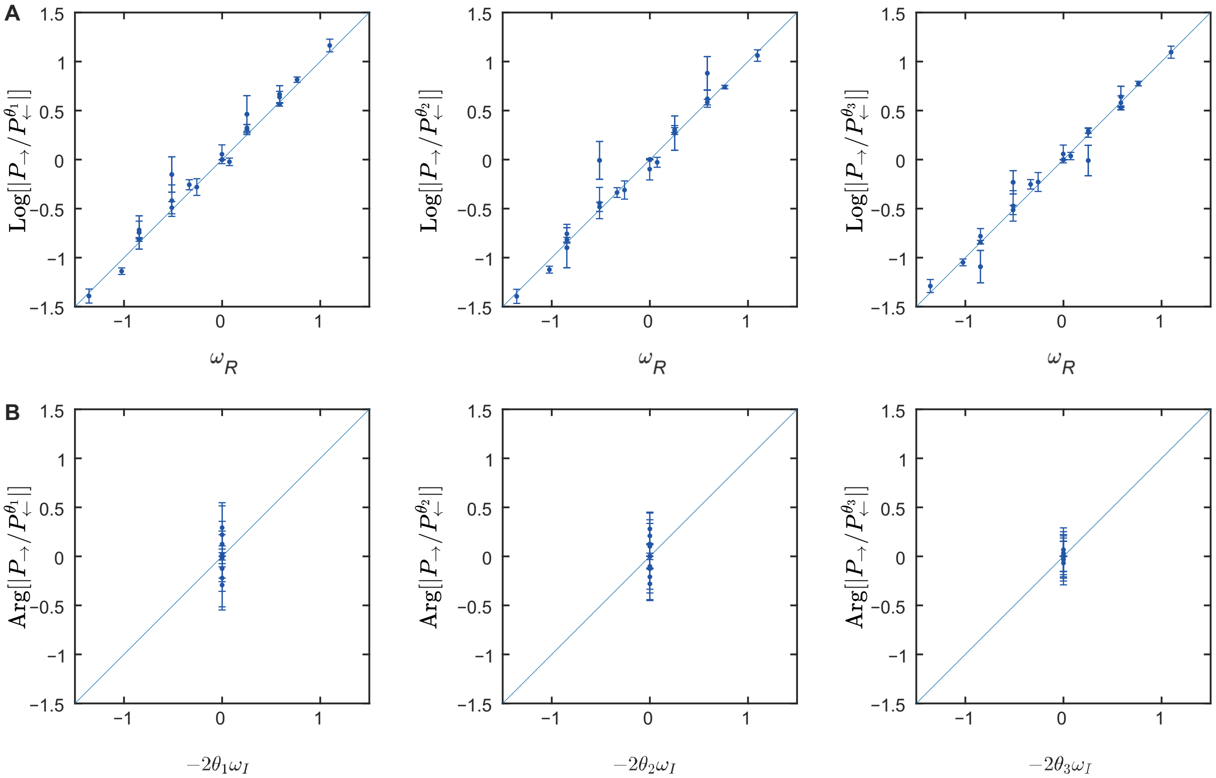

Fig. S9. Experimental verification of generalized quantum Crooks FT for a covariant channel. **(A)** and **(B)** correspond to the real and imaginary parts of the quasi-probability ratio, respectively. The dashed line are the theoretical prediction of the log-magnitude and the phase of the quasi-probability ratio. Experimental data are plotted with error bars.

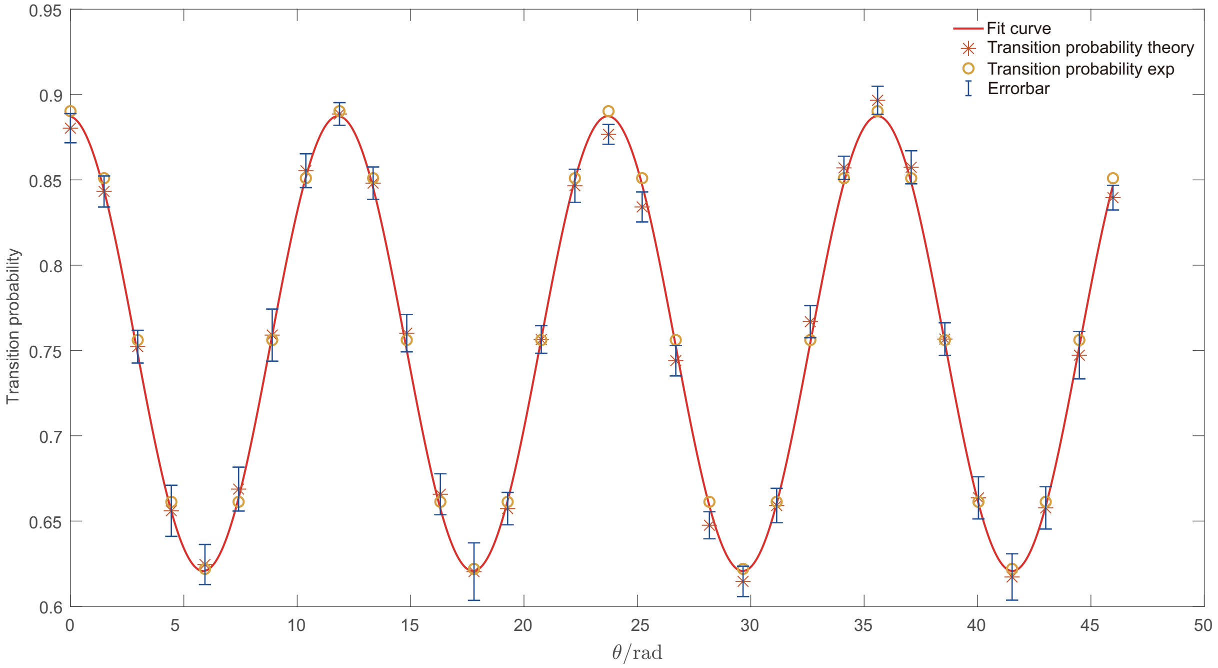

Fig. S10. Experimental results of transition probability between the rotated eigenstates $e^{i\theta \log \hat{\gamma}} |\psi_0\rangle$ and $e^{i\theta \log \hat{\gamma}} |\phi'_{0'}\rangle$. Yellow circles represent the experimental data, the red asterisk stands for the theoretical transition probability value and the red line corresponds to the fitted function. Experimental data are plotted with error bars.

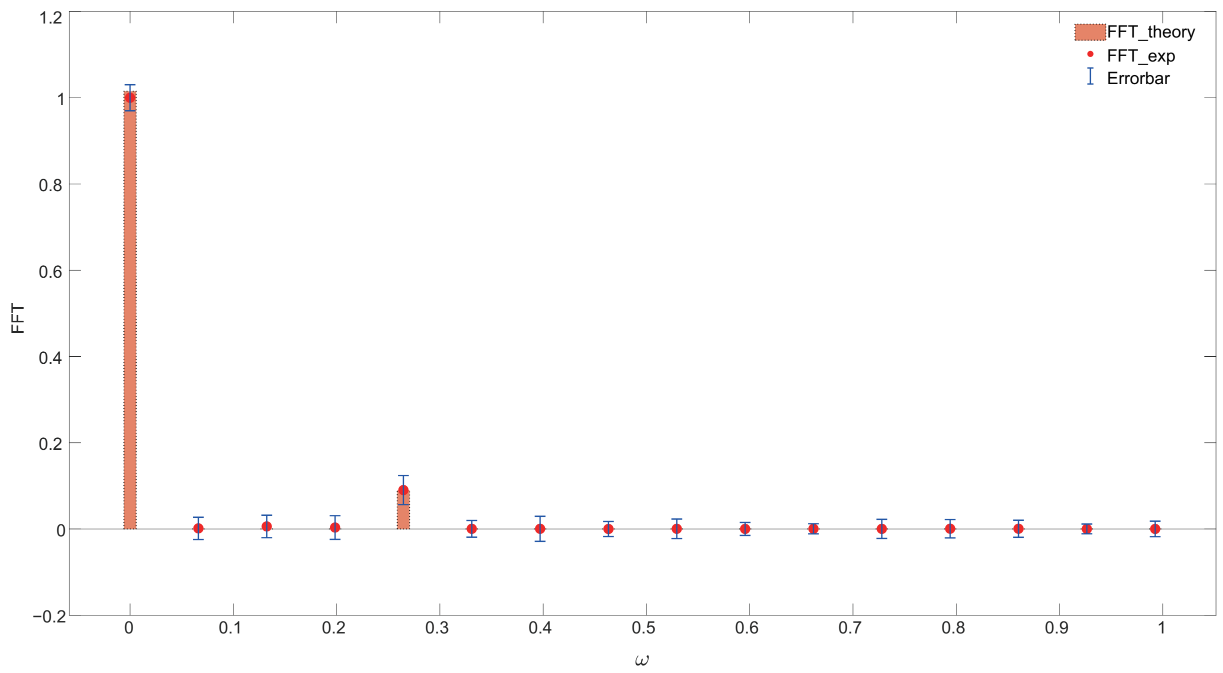

Fig. S11. Normalized fast Fourier transformation (FFT) of the experimentally derived discrete transition probabilities. The red point are the experimental data and the orange circle are the theoretical predication. Experimental data are plotted with error bars.

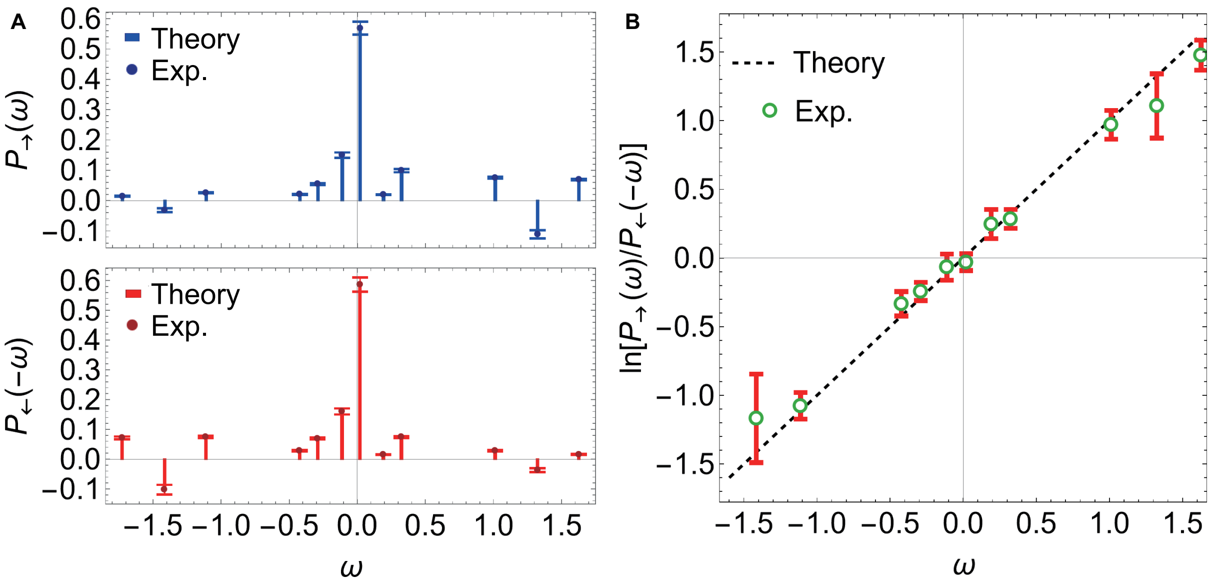

Fig. S12. Reconstructed quasi-probability distributions and the QFT when $\alpha = \pi/4$. (A), Theoretically predicted (bars) and experimentally reconstructed quasi-probability distributions (points) for the forward process $P_{\rightarrow}$ (blue) and time-reversal process $P_{\leftarrow}$ (red) of quantum entropy production ω when $\alpha = \pi/4$. **(B)**, The ratio between the forward and backward processes, respectively. Experimental data are plotted with error bars.

Table S1. The deviations of the experimentally reconstructed quasi-probabilities from the theory.

Channel	Process	Value
$\mathcal{N}$	$\sum_\omega \lvert P_{\rightarrow}^{\text{exp.}}(\omega) - P_{\rightarrow}^{\text{theory}}(\omega)\rvert$	0.0459 ± 0.0231
$\mathcal{N}$	$\sum_\omega \lvert P_{\leftarrow}^{\text{exp.}}(\omega) - P_{\leftarrow}^{\text{theory}}(\omega)\rvert$	0.0734 ± 0.0136
$\mathcal{N}_{\text{cov}}$	$\sum_\omega \lvert P_{\rightarrow}^{\text{exp.}}(\omega) - P_{\rightarrow}^{\text{theory}}(\omega)\rvert$	0.0330 ± 0.0137
$\mathcal{N}_{\text{cov}}$	$\sum_\omega \lvert P_{\leftarrow}^{\text{exp.}}(\omega) - P_{\leftarrow}^{\text{theory}}(\omega)\rvert$	0.0476 ± 0.0134

REFERENCES AND NOTES

1. G. E. Crooks, Entropy production fluctuation theorem and the nonequilibrium work relation for free energy differences. *Phys. Rev. E* **60**, 2721–2726 (1999).

2. U. Seifert, Entropy production along a stochastic trajectory and an integral fluctuation theorem. *Phys. Rev. Lett.* **95**, 040602 (2005).

3. C. Jarzynski, Nonequilibrium equality for free energy differences. *Phys. Rev. Lett.* **78**, 2690–2693 (1997).

4. M. Esposito, U. Harbola, S. Mukamel, Nonequilibrium fluctuations, fluctuation theorems, and counting statistics in quantum systems. *Rev. Mod. Phys.* **81**, 1665–1702 (2009).

5. M. Campisi, P. Hänggi, P. Talkner, Colloquium: Quantum fluctuation relations: Foundations and applications. *Rev. Mod. Phys.* **83**, 771–791 (2011).

6. J. Liphardt, S. Dumont, S. B. Smith, I. Tinoco, C. Bustamante, Equilibrium information from nonequilibrium measurements in an experimental test of Jarzynski's equality. *Science* **296**, 1832–1835 (2002).

7. D. Collin, F. Ritort, C. Jarzynski, S. B. Smith, I. Tinoco, C. Bustamante, Verification of the Crooks fluctuation theorem and recovery of RNA folding free energies. *Nature* **437**, 231–234 (2005).

8. V. Blickle, T. Speck, L. Helden, U. Seifert, C. Bechinger, Thermodynamics of a colloidal particle in a time-dependent nonharmonic potential. *Phys. Rev. Lett.* **96**, 070603 (2006).

9. N. C. Harris, Y. Song, C.-H. Kiang, Experimental free energy surface reconstruction from single-molecule force spectroscopy using Jarzynski's equality. *Phys. Rev. Lett.* **99**, 068101 (2007).

10. S. Toyabe, T. Sagawa, M. Ueda, E. Muneyuki, M. Sano, Experimental demonstration of information-to-energy conversion and validation of the generalized Jarzynski equality. *Nat. Phys.* **6**, 988–992 (2010).

11. O.-P. Saira, Y. Yoon, T. Tanttu, M. Möttönen, D. V. Averin, J. P. Pekola, Test of the Jarzynski and Crooks fluctuation relations in an electronic system. *Phys. Rev. Lett.* **109**, 180601 (2012).

12. P. Talkner, E. Lutz, P. Hänggi, Fluctuation theorems: Work is not an observable. *Phys. Rev. E* **75**, 050102 (2007).

13. G. T. Landi, M. Paternostro, Irreversible entropy production: From classical to quantum. *Rev. Mod. Phys.* **93**, 035008 (2021).

14. G. Huber, F. Schmidt-Kaler, S. Deffner, E. Lutz, Employing trapped cold ions to verify the quantum Jarzynski equality. *Phys. Rev. Lett.* **101**, 070403 (2008).

15. S. An, J.-N. Zhang, M. Um, D. Lv, Y. Lu, J. Zhang, Z.-Q. Yin, H. T. Quan, K. Kim, Experimental test of the quantum Jarzynski equality with a trapped-ion system. *Nat. Phys.* **11**, 193–199 (2015).

16. A. Smith, Y. Lu, S. An, X. Zhang, J.-N. Zhang, Z. Gong, H. T. Quan, C. Jarzynski, K. Kim, Verification of the quantum nonequilibrium work relation in the presence of decoherence. *New J. Phys.* **20**, 013008 (2018).

17. T. B. Batalhão, A. M. Souza, L. Mazzola, R. Auccaise, R. S. Sarthour, I. S. Oliveira, J. Goold, G. De Chiara, M. Paternostro, R. M. Serra, Experimental reconstruction of work distribution and study of fluctuation relations in a closed quantum system. *Phys. Rev. Lett.* **113**, 140601 (2014).

18. F. Cerisola, Y. Margalit, S. Machluf, A. J. Roncaglia, J. P. Paz, R. Folman, Using a quantum work meter to test non-equilibrium fluctuation theorems. *Nat. Commun.* **8**, 1241 (2017).

19. Z. Zhang, T. Wang, L. Xiang, Z. Jia, P. Duan, W. Cai, Z. Zhan, Z. Zong, J. Wu, L. Sun, Y. Yin, G. Guo, Experimental demonstration of work fluctuations along a shortcut to adiabaticity with a superconducting Xmon qubit. *New J. Phys.* **20**, 085001 (2018).

20. A. Solfanelli, A. Santini, M. Campisi, Experimental verification of fluctuation relations with a quantum computer. *PRX Quantum* **2**, 030353 (2021).

21. G. H. Aguilar, T. L. Silva, T. E. Guimarães, R. S. Piera, L. C. Céleri, G. T. Landi, Two-point measurement of entropy production from the outcomes of a single experiment with correlated photon pairs. *Phys. Rev. A* **106**, L020201 (2022).

22. M. Bellini, H. Kwon, N. Biagi, S. Francesconi, A. Zavatta, M. S. Kim, Demonstrating quantum microscopic reversibility using coherent states of light. *Phys. Rev. Lett.* **129**, 170604 (2022).

23. A. E. Allahverdyan, Nonequilibrium quantum fluctuations of work. *Phys. Rev. E* **90**, 032137 (2014).

24. M. Perarnau-Llobet, E. Bäumer, K. V. Hovhannisyan, M. Huber, A. Acin, No-go theorem for the characterization of work fluctuations in coherent quantum systems. *Phys. Rev. Lett.* **118**, 070601 (2017).

25. K.-D. Wu, Y. Yuan, G.-Y. Xiang, C.-F. Li, G.-C. Guo, M. Perarnau-Llobet, Experimentally reducing the quantum measurement back action in work distributions by a collective measurement. *Sci. Adv.* **5**, eaav4944 (2019).

26. P. Solinas, S. Gasparinetti, Full distribution of work done on a quantum system for arbitrary initial states. *Phys. Rev. E* **92**, 042150 (2015).

27. P. Solinas, M. Amico, N. Zanghì, Measurement of work and heat in the classical and quantum regimes. *Phys. Rev. A* **103**, L060202 (2021).

28. B.-M. Xu, J. Zou, L.-S. Guo, X.-M. Kong, Effects of quantum coherence on work statistics. *Phys. Rev. A* **97**, 052122 (2018).

29. C. L. Latune, I. Sinayskiy, F. Petruccione, Negative contributions to entropy production induced by quantum coherences. *Phys. Rev. A* **102**, 042220 (2020).

30. J. P. Santos, L. C. Céleri, G. T. Landi, M. Paternostro, The role of quantum coherence in non-equilibrium entropy production. *NPJ Quantum Inf.* **5**, 1–7 (2019).

31. S. Jevtic, T. Rudolph, D. Jennings, Y. Hirono, S. Nakayama, M. Murao, Exchange fluctuation theorem for correlated quantum systems. *Phys. Rev. E* **92**, 042113 (2015).

32. K. Micadei, G. T. Landi, E. Lutz, Quantum fluctuation theorems beyond two-point measurements. *Phys. Rev. Lett.* **124**, 090602 (2020).

33. J. Åberg, Fully quantum fluctuation theorems. *Phys. Rev. X* **8**, 011019 (2018).

34. Z. Holmes, S. Weidt, D. Jennings, J. Anders, F. Mintert, Coherent fluctuation relations: From the abstract to the concrete. *Quantum* **3**, 124 (2019).

35. T. Hatano, S.-I. Sasa, Steady-state thermodynamics of Langevin systems. *Phys. Rev. Lett.* **86**, 3463–3466 (2001).

36. M. Esposito, C. Van den Broeck, Three detailed fluctuation theorems. *Phys. Rev. Lett.* **104**, 090601 (2010).

37. G. Manzano, J. M. Horowitz, J. M. R. Parrondo, Quantum fluctuation theorems for arbitrary environments: Adiabatic and nonadiabatic entropy production. *Phys. Rev. X* **8**, 031037 (2018).

38. K. Micadei, J. P. S. Peterson, A. M. Souza, R. S. Sarthour, I. S. Oliveira, G. T. Landi, R. M. Serra, E. Lutz, Experimental validation of fully quantum fluctuation theorems using dynamic Bayesian networks. *Phys. Rev. Lett.* **127**, 180603 (2021).

39. S. Hernández-Gómez, S. Gherardini, A. Belenchia, A. Trombettoni, M. Paternostro, N. Fabbri, Experimental signature of initial quantum coherence on entropy production. *NPJ Quantum Inf.* **9**, 86 (2023).

40. F. L. Rodrigues, E. Lutz, Nonequilibrium thermodynamics of quantum coherence beyond linear response. *Commun. Phys.* **7**, 61 (2024).

41. H. Kwon, M. S. Kim, Fluctuation theorems for a quantum channel. *Phys. Rev. X* **9**, 031029 (2019).

42. J. G. Kirkwood, Quantum statistics of almost classical assemblies. *Phys. Rev.* **44**, 31–37 (1933).

43. P. A. M. Dirac, On the analogy between classical and quantum mechanics. *Rev. Mod. Phys.* **17**, 195–199 (1945).

44. M. Lostaglio, Quantum fluctuation theorems, contextuality, and work quasiprobabilities. *Phys. Rev. Lett.* **120**, 040602 (2018).

45. A. Levy, M. Lostaglio, Quasiprobability distribution for heat fluctuations in the quantum regime. *PRX Quantum* **1**, 010309 (2020).

46. K. Zhang, J. Wang, Quasiprobability fluctuation theorem behind the spread of quantum information. *Commun. Phys.* **7**, 91 (2024).

47. M. Lostaglio, A. Belenchia, A. Levy, S. Hernández-Gómez, N. Fabbri, S. Gherardini, Kirkwood-Dirac quasiprobability approach to the statistics of incompatible observables. *Quantum* **7**, 1128 (2023).

48. D. Petz, Sufficient subalgebras and the relative entropy of states of a von neumann algebra. *Commun. Math. Phys.* **105**, 123–131 (1986).

49. M. Junge, R. Renner, D. Sutter, M. M. Wilde, A. Winter, Universal recovery maps and approximate sufficiency of quantum relative entropy. *Ann. Henri Poincaré* **19**, 2955–2978 (2018).

50. M. D. Vidrighin, O. Dahlsten, M. Barbieri, M. S. Kim, V. Vedral, I. A. Walmsley, Photonic Maxwell's demon. *Phys. Rev. Lett.* **116**, 050401 (2016).

51. L. Mancino, M. Sbroscia, I. Gianani, E. Roccia, M. Barbieri, Quantum simulation of single-qubit thermometry using linear optics. *Phys. Rev. Lett.* **118**, 130502 (2017).

52. L. Mancino, V. Cavina, A. De Pasquale, M. Sbroscia, R. I. Booth, E. Roccia, I. Gianani, V. Giovannetti, M. Barbieri, Geometrical bounds on irreversibility in open quantum systems. *Phys. Rev. Lett.* **121**, 160602 (2018).

53. W. Tham, H. Ferretti, A. Sadashivan, A. Steinberg, Simulating and optimising quantum thermometry using single photons. *Sci. Rep.* **6**, 38822 (2016).

54. R. M. de Araújo, T. Häffner, R. Bernardi, D. S. Tasca, M. P. J. Lavery, M. J. Padgett, A. Kanaan, L. C. Céleri, P. H. S. Ribeiro, Experimental study of quantum thermodynamics using optical vortices. *J. Phys. Commun.* **2**, 035012 (2018).

55. G. E. Crooks, Quantum operation time reversal. *Phys. Rev. A* **77**, 034101 (2008).

56. L. Hu, X. Mu, W. Cai, Y. Ma, Y. Xu, H. Wang, Y. Song, C.-L. Zou, L. Sun, Experimental repetitive quantum channel simulation. *Sci. Bull.* **63**, 1551–1557 (2018).

57. H. Lu, C. Liu, D.-S. Wang, L.-K. Chen, Z.-D. Li, X.-C. Yao, L. Li, N.-L. Liu, C.-Z. Peng, B. C. Sanders, Y.-A. Chen, J.-W. Pan, Experimental quantum channel simulation. *Phys. Rev. A* **95**, 042310 (2017).

58. K. Wang, X. Wang, X. Zhan, Z. Bian, J. Li, B. C. Sanders, P. Xue, Entanglement-enhanced quantum metrology in a noisy environment. *Phys. Rev. A* **97**, 042112 (2018).

59. P. Wang, H. Kwon, C.-Y. Luan, W. Chen, M. Qiao, Z. Zhou, K. Wang, M. S. Kim, K. Kim, Snapshotting quantum dynamics at multiple time points. *Nat. Commun.* **15**, 8900 (2024).

60. J. S. Lundeen, C. Bamber, Procedure for direct measurement of general quantum states using weak measurement. *Phys. Rev. Lett.* **108**, 070402 (2012).

61. S. Gherardini, M. M. Müller, A. Trombettoni, S. Ruffo, F. Caruso, Reconstructing quantum entropy production to probe irreversibility and correlations. *Quantum Sci. Technol.* **3**, 035013 (2018).

62. D. R. Arvidsson-Shukur, W. F. Braasch Jr., S. De Bievre, J. Dressel, A. N. Jordan, C. Langrenez, M. Lostaglio, J. S. Lundeen, N. Y. Halpern, Properties and applications of the Kirkwood-Dirac distribution. *New J. Phys.* **26**, 121201 (2024).

63. A. Gilyén, S. Lloyd, I. Marvian, Y. Quek, M. M. Wilde, Quantum algorithm for Petz recovery channels and pretty good measurements. *Phys. Rev. Lett.* **128**, 220502 (2022).

64. H. Barnum, E. Knill, Reversing quantum dynamics with near-optimal quantum and classical fidelity. *J. Math. Phys.* **43**, 2097–2106 (2002).

65. J. M. Gertler, B. Baker, J. Li, S. Shirol, J. Koch, C. Wang, Protecting a bosonic qubit with autonomous quantum error correction. *Nature* **590**, 243–248 (2021).

66. H. Kwon, R. Mukherjee, M. S. Kim, Reversing Lindblad dynamics via continuous Petz recovery map. *Phys. Rev. Lett.* **128**, 020403 (2022).

67. F. Brandão, M. Horodecki, N. Ng, J. Oppenheim, S. Wehner, The second laws of quantum thermodynamics. *Proc. Natl. Acad. Sci. U.S.A.* **112**, 3275–3279 (2015).

68. B. P. Lanyon, M. Barbieri, M. P. Almeida, T. Jennewein, T. C. Ralph, K. J. Resch, G. J. Pryde, J. L. O'brien, A. Gilchrist, A. G. White, Simplifying quantum logic using higher-dimensional hilbert spaces. *Nat. Phys.* **5**, 134–140 (2009).

69. Z. Hou, G. Xiang, D. Dong, C.-F. Li, G.-C. Guo, Realization of mutually unbiased bases for a qubit with only one wave plate: Theory and experiment. *Opt. Express* **23**, 10018–10031 (2015).

70. M. G. Paris, The modern tools of quantum mechanics: A tutorial on quantum states, measurements, and operations. *Eur. Phys. J. Spec. Top.* **203**, 61–86 (2012).

71. I. L. Chuang, M. A. Nielsen, Prescription for experimental determination of the dynamics of a quantum black box. *J. Mod. Opt.* **44**, 2455–2467 (1997).
